# Canine Pyometra: A Short Review of Current Advances

**DOI:** 10.3390/ani13213310

**Published:** 2023-10-25

**Authors:** Rafael Gariglio Clark Xavier, Clarissa Helena Santana, Yasmin Gonçalves de Castro, Thayanne Gabryelle Viana de Souza, Victor Santos do Amarante, Renato Lima Santos, Rodrigo Otávio Silveira Silva

**Affiliations:** Veterinary School, Federal University of Minas Gerais, Antônio Carlos Avenue 6627, Belo Horizonte 31270-090, Brazil

**Keywords:** reproductive, *Escherichia coli*, uterine

## Abstract

**Simple Summary:**

Pyometra is a common reproductive disease in dogs that often begins with mild symptoms, but if not promptly treated, it can turn into a threat to life. Despite being frequent, the disease is still not fully understood. In the last few years, studies have contributed to a better comprehension of this disease, raising new hypotheses regarding the epidemiology, bacteria involved, the pre-existing uterine lesions that might be associated, and even a possible influence of one’s diet. In light of this, this work aimed to review the current understanding of canine pyometra, with particular emphasis on the recent research findings.

**Abstract:**

Pyometra, characterized by the accumulation of purulent exudate in the uterus, is the most prevalent reproductive disease in canines. While the disease often begins with mild local symptoms, it can escalate into peritonitis, sepsis, and multi-organ dysfunction, thereby posing a significant threat to life. Despite the high incidence and recognized significance of canine pyometra, gaps persist in our understanding of its epidemiology, etiology, and pathogenesis. Recent studies have, however, broadened our comprehension of this disease, shedding light on potential new infection sources, etiologies, and the application of clinical predictive biomarkers and new therapeutic protocols. This study aimed to review the current understanding of canine pyometra, with particular emphasis on the latest research concerning its etiology and epidemiology. Furthermore, it addressed key research questions and proposed directions for future investigations into various facets of canine pyometra.

## 1. Introduction

Pyometra is characterized by the accumulation of purulent exudate in the uterine lumen and is the most prevalent reproductive disease in canines [1]. It typically develops during the luteal phase, with *Escherichia coli* being the most frequently isolated bacteria [2,3,4]. Other commonly reported microorganisms include *Staphylococcus pseudintermedius* and *Streptococcus canis*. Recent studies, however, have suggested the potential involvement of less common pathogens, including *Brucella abortus*, *Corynebacterium* spp., and possibly *Porphyromonas* spp. [5,6].

Canine pyometra typically begins with subtle clinical signs such as polydipsia, polyuria, and vaginal discharge. Without timely treatment, it can progress to peritonitis, sepsis, and the dysfunction of multiple organs [7,8]. Consequently, it is regarded as a life-threatening infection [9,10,11].

Despite the prevalent occurrence and recognized significance of canine pyometra, our understanding of its epidemiology, etiology, and pathogenesis remains incomplete. Recent studies have broadened our knowledge of this disease, identifying potential new infection sources, causes, and biomarkers that could aid in predicting its prognosis and severity. Consequently, this review aimed to consolidate the current knowledge on canine pyometra, with particular emphasis on the latest research concerning its etiology and epidemiology.

## 2. Epidemiology and Risk Factors

**Figure 1:**
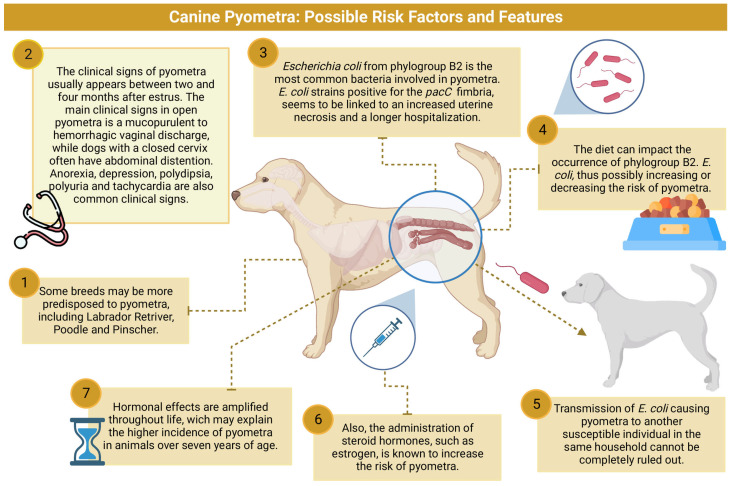
Infographic summarizing the possible risk factors and features of canine pyometra. 1—Some breeds may be more predisposed to pyometra [13,16,20]. 2—Although pyometra is primarily caused by microorganisms from the gastrointestinal tract, recent studies have suggested that other sources, including those that are hematogenous, are potential contributors [5,6]. 3—The most common bacteria involved in pyometra is phylogroup B2 *E. coli*, which ascend from the rectum microbiota to the uterus [21,22,23]. 4—Diet seems to impact the frequency of phylogroup B2 *E. coli* [21]. 5—Transmission of *E. coli*-causing-pyometra to another susceptible individual in the same household was recently reported [22]. 6—Administration of steroid hormones increases the risk of pyometra [23,24,25,26]. 7—A higher occurrence of pyometra is seen in animals around seven years of age, but the disease has been described in animals ranging in age from three months to 20 years [14,15,16,23]. Created using BioRender^®^ (https://www.biorender.com/).

Pyometra, a bacterial infection in the uterus, is the most prevalent reproductive disease in dogs, impacting up to 25% of non-castrated females [1]. This disease is characterized by a bacterial infection in the uterus that results in local and systemic clinical signs [10,12,13]. Although pyometra can occur in dogs ranging from 3 months to 20 years old, it predominantly affects middle-aged to older dogs (Figure 1), with a median diagnosis age of nine years [14,15,16]. The higher incidence of pyometra in middle-aged to older dogs is thought to be associated with repeated estrous cycles. During diestrus, progesterone enhances the secretory activity of the endometrial glands, promotes endometrial proliferation, diminishes myometrium contractility, and induces cervix closure [17]. Additionally, diestrus also reduces local leukocyte responses and uterine resistance to bacterial infection [1,18]. These effects, which accumulate after repeated estrous cycles, escalate the risk of pyometra with each cycle [14,17,19].

Some studies suggest that certain breeds may be more susceptible to pyometra (Table 1) [13,14,27,28]. However, the prevalence of pyometra appears to fluctuate according to studies conducted across various countries, and the hypothesis of breed predisposition remains speculative. Recent research involving Golden Retrievers has identified a potential correlation between pyometra and specific changes in the ABCC4 gene located on chromosome 22 [20,23]. This discovery introduces, for the first time, a potential explanation for the increased incidence of pyometra in a particular breed. Despite this finding, there remains no definitive evidence of breed predisposition to pyometra, and the reasons for its higher prevalence in some breeds largely remain a mystery.

The administration of drugs used for reproductive control, such as progestogens or estrogen compounds, is a recognized predisposing factor for canine pyometra [24,25,26,32]. These drugs, which suppress the sexual receptivity phase in female dogs, have been linked to an increased risk of pyometra and other conditions, including fetal maceration, endometrial and mammary tumors, and insulin resistance [26,33,34]. Hormonal effects, which intensify over time, may account for the higher incidence of pyometra in animals over seven years of age [15,23].

## 3. Etiopathogenesis

**Figure 2:**
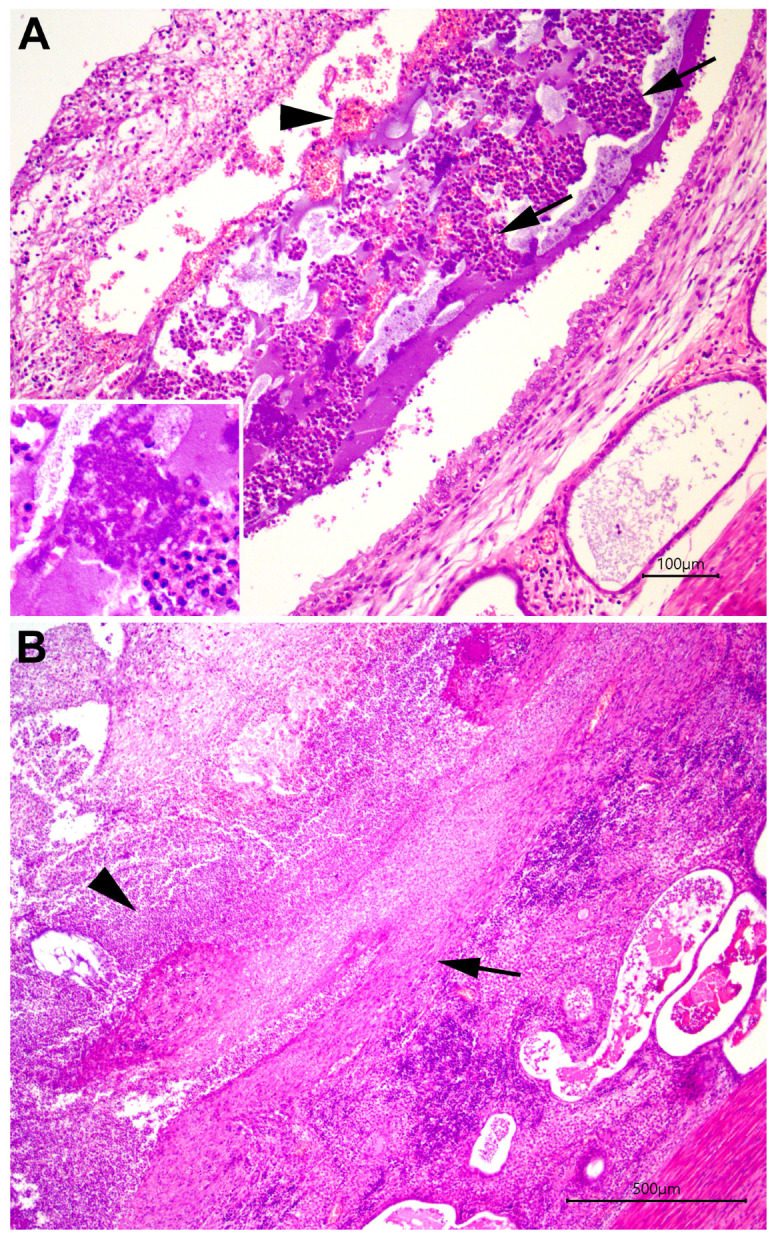
Uterus from a female dog with pyometra. (**A**) Endometrium with diffuse severe neutrophilic inflammatory infiltrates (arrows); hemorrhage (arrowhead), fibrin, and intraluminal bacterial aggregates (inset); and a columnar and vacuolated endometrial superficial epithelium (decidual reaction) and ectasia of endometrial glands in a case of pseudo-placentacional endometrial hyperplasia. HE; bar = 100 μm. (**B**) Endometrium with necrosis and superficial epithelial loss (arrow), with a diffuse severe neutrophilic inflammatory (arrowhead) infiltrate and mild hemorrhage. Endometrium with diffuse severe interstitial lymphoplasmacytic inflammation and marked glandular ectasia. HE; bar = 500 μm.

Despite the high incidence of canine pyometra, its pathogenesis remains inadequately understood. It is evident, however, that this pathogenesis is multifactorial, involving bacterial infection, hormonal changes (or a favorable endocrine environment), genetic predisposition, and pre-existing uterine lesions [35]. During the luteal phase of the estrous cycle (diestrus), progesterone stimulates the proliferation and secretion of endometrial glands. Moreover, progesterone inhibits myometrial contraction and weakens the uterine immune response, thereby promoting bacterial colonization [10,13,36]. Early studies on the pathogenesis of canine pyometra established a connection between hormonal stimulation and the occurrence of pyometra [32]. At that time, cystic endometrial hyperplasia was considered a predisposing endometrial lesion leading to pyometra under experimental conditions [32]. However, it was later discovered that, in addition to cystic endometrial hyperplasia, bitches in diestrus often develop another type of proliferative change in the endometrium. This change is characterized by endometrial hyperplasia with glandular cystic changes and decidual changes affecting the superficial endometrial epithelium, termed “pseudoplacentational endometrial hyperplasia” (Figure 2A,B) [37]. A recent study showed that in naturally occurring canine pyometra, pseudoplacentational endometrial hyperplasia is significantly associated with pyometra, whereas cystic endometrial hyperplasia is not [38]. Notably, despite this significant association, a cause-and-effect relationship between pseudoplacentational endometrial hyperplasia and pyometra has yet to be established [23,38]. These recent findings [38] suggest that the traditional terminology of the “cystic endometrial hyperplasia-pyometra complex” is outdated [35]. However, this should not be misinterpreted as diminishing the importance of endometrial hyperplastic changes in the pathogenesis of canine pyometra.

A broad spectrum of bacteria can contribute to pyometra in dogs [6,39]. *E. coli* is among the most prevalent microorganisms, implicated in up to 90% of canine pyometra cases (Table 2). This Gram-negative facultative anaerobic bacterium is also the primary pathogen in uterine infections across various species, including humans [4,40,41]. As *E. coli* is a component of the gut microbiota, it has been postulated that this microorganism can ascend from the rectum to the uterus, thereby causing this disease. This theory has been substantiated by studies demonstrating that the *E. coli* strains responsible for pyometra are often indistinguishable from those colonizing the gastrointestinal tract of the same dog [9,22,42]. Intriguingly, most dogs with pyometra are gut-colonized specifically by *E. coli* from phylogroup B2 [21], the same phylogroup frequently isolated from the uterine contents of affected animals [21,43,44]. Conversely, healthy dogs are more commonly gut-colonized by other phylogroups, including B1 [21,43,45]. This observation has led to the hypothesis that colonization by certain *E. coli* strains may elevate the risk of pyometra. In this context, a recent study demonstrated that diet can influence the colonization rate by *E. coli* from phylogroup B2 in the gut, suggesting that certain diets may indirectly heighten the risk of pyometra. If this hypothesis is further validated, strategies for altering or modulating the microbiota could provide an additional means of preventing or reducing the risk of pyometra [21].

In addition to phylogroup studies, researchers have examined the presence of virulence factors in *E. coli* isolated from canine pyometra. Some suggest that the possession of a specific combination of virulence genes may determine the severity of pyometra in female dogs [44,46]. Among these virulence factors, the gene encoding type P fimbriae (*papC*) has recently attracted considerable attention. Firstly, the prevalence of this gene is often higher in *E. coli* isolates from dogs with pyometra (ranging between 36.5 and 44.1%) compared to strains from the gut of healthy dogs (ranging between 18.2 and 29.2%) [21,47]. Secondly, experimental studies have shown that this fimbria plays a crucial role in the adhesion and colonization of *E. coli* in the canine endometrium [48]. A recent study also revealed a higher degree of uterine necrosis in dogs with pyometra caused by *E. coli papC*-positive strains. Interestingly, the degree of necrosis was positively correlated with the duration of hospitalization, suggesting a potential link between this fimbria and disease severity [16]. Another study proposed that uterine *E. coli* infection could alter the expression of sex hormone receptors in the uterus of female dogs, thereby enhancing the hormonal factors that promote bacterial growth [49]. Collectively, these studies strongly suggest that certain *E. coli* strains, possessing specific virulence traits, may be more likely to cause canine pyometra by facilitating tissue colonization and even modifying the uterine environment so that infection is favored. Further research is required to better understand the influence of the gut microbiota and diet on colonization by pyometra-causing *E. coli*.

In addition to *E. coli*, other members of the Enterobacteriaceae family, such as *Klebsiella pneumoniae* and *Proteus mirabilis*, are frequently implicated in pyometra. Bacteria from the *Streptococcus*, *Staphylococcus*, and *Enterococcus* genera are also noteworthy (Table 2). Studies have demonstrated that, similar to *E. coli*, *K. pneumoniae*, *S. pseudintermedius*, *S. canis*, and *E. faecalis* strains isolated from dogs with pyometra differ from most commensal strains. They express virulence factors such as adhesins, toxins, iron acquisition mechanisms, and mechanisms for evading the host immune system. These factors facilitate colonization and sustain the infection in the canine uterus [47,50,51,52,53].

Studies have intriguingly reported that no microorganisms are isolated in up to 25% of pyometra cases [8,21,54]. Several hypotheses have been proposed to explain this phenomenon, including the host immune system’s elimination of the pathogen, the use of antimicrobials during the preoperative period, the low sensitivity of culture methods, and the existence of microorganisms that do not grow in the standard culture media used for routine diagnosis [54]. This last hypothesis has been reinforced by studies that have identified the presence of some uncommon microorganisms causing pyometra, such as *Mycoplasma* spp., *Nocardia* spp., *Corynebacterium* spp., *Moraxella* spp., *Clostridium perfringens*, *Porphyromonas* spp., and *Brucella abortus* [5,6]. While the infection in most cases likely ascends from the gastrointestinal tract, the detection of certain specific bacteria, including *Brucella abortus*, suggests that other infection routes, such as hematogenous pathways, are also possible [9,42,55]. Notably, *Porphyromonas* sp. has recently been confirmed as a cause of pyometra, leading to the hypothesis that bacteria typically found in the oral cavity can cause pyometra. Interestingly, *Porphyromonas* sp. is a well-established cause of reproductive diseases in humans as well as endocarditis, lung, liver, and kidney infections, which can spread through the bloodstream (hematogenously) [56,57,58,59,60].

## 4. Clinical Presentation

**Figure 3:**
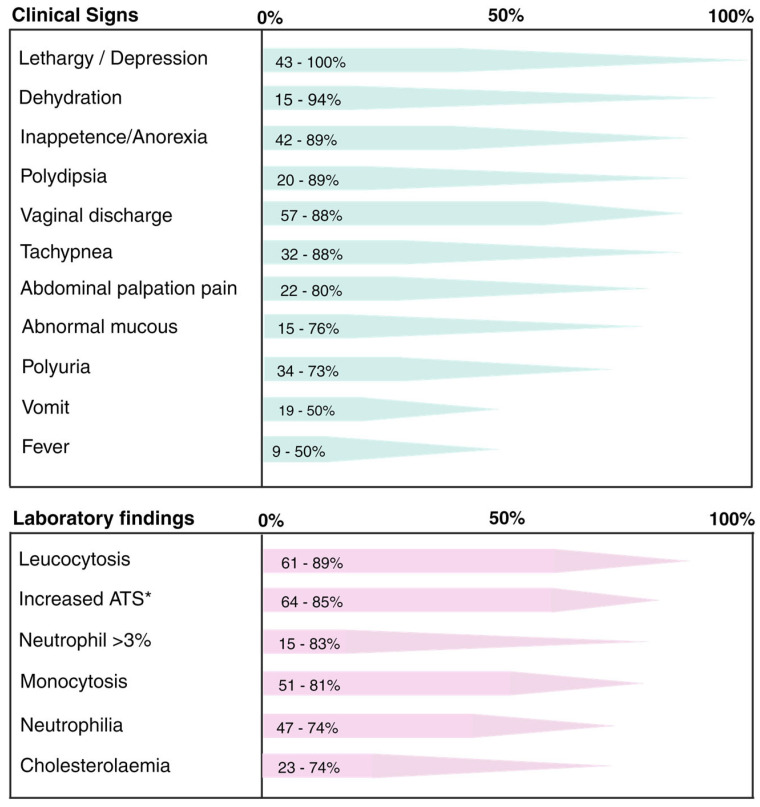
Main clinical signs and laboratory findings in female dogs with pyometra, according to previous reports [7,11,16,23,63,64,65,66]. * AST—Aspartate aminotransferase.

**Figure 4:**
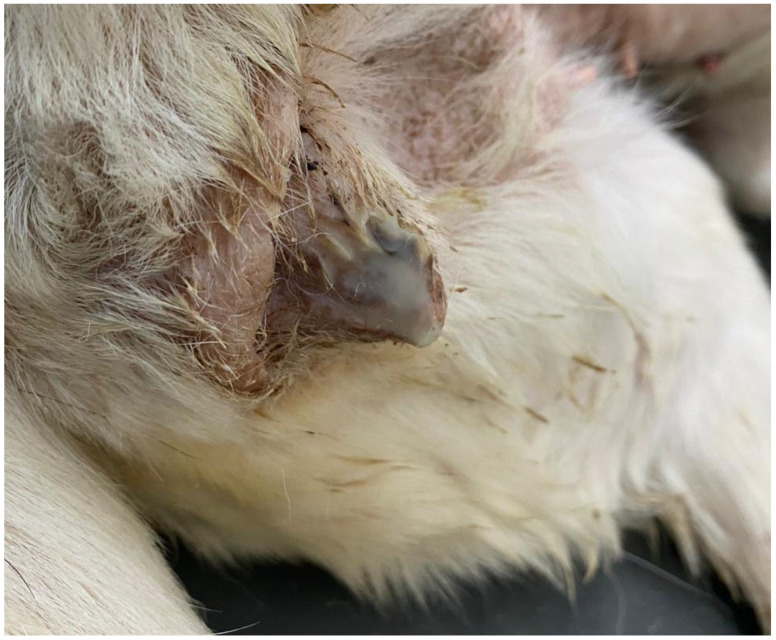
Purulent vaginal discharge in a bitch with open cervix pyometra.

**Figure 5:**
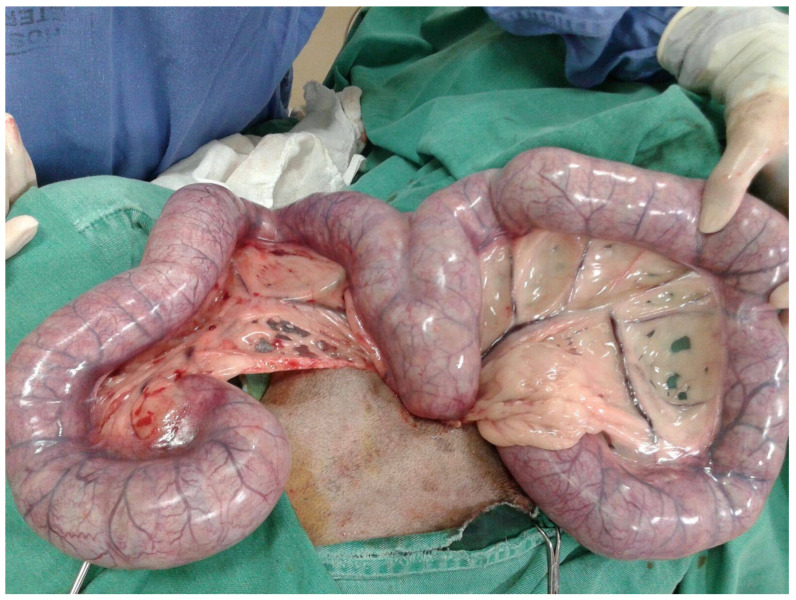
Intraoperative image of an enlarged, pus-filled uterus in a bitch (mixed-breed dog) with pyometra.

Pyometra typically manifests with local and systemic clinical signs (Figure 3), generally appearing between two and four-months post-estrus [9,10,61]. The most prevalent clinical symptom in dogs with open pyometra is the presence of vaginal discharge that ranges from mucopurulent to hemorrhagic in nature (Figure 4) [17]. Conversely, dogs with a closed cervix often exhibit abdominal distention owing to the lack of uterine content drainage (Figure 5) [7]. 

Clinical findings in pyometra cases can vary, but they commonly include inappetence/anorexia, depression/lethargy, polydipsia, polyuria, tachycardia, and tachypnea [8,11,17]. Pyometra is a life-threatening condition because of the potential for complications such as uterine rupture, nephropathy, peritonitis, endotoxemia, and, particularly, sepsis [13,38,62].

Notably, fever and hypothermia have been identified as factors increasing the risk of peritonitis development. Concurrently, moderate to severe general depression and pale mucous membranes are linked to extended hospitalization periods [11]. Furthermore, animals with closed pyometra exhibit a more severe condition and an elevated risk of sepsis [7,67].

## 5. Diagnosis

The clinical diagnosis of this disease is often made for cases of open pyometra. However, in the absence of vaginal discharge, diagnosis can be significantly more challenging owing to the variability of other clinical signs [8]. Typically, diagnosis relies on patient history, clinical signs, and imaging tests such as abdominal radiography and ultrasound. Additional tests, including blood counts, leukograms, and liver function evaluations, can also provide valuable information (Figure 3) [11,13,44]. Leukocytosis and anemia, along with signs of azotemia, are frequently observed in affected animals. This is because renal dysfunction can result from endotoxemia, glomerular dysfunction, renal tubular damage, and a decreased response to the antidiuretic hormone [8].

**Figure 6:**
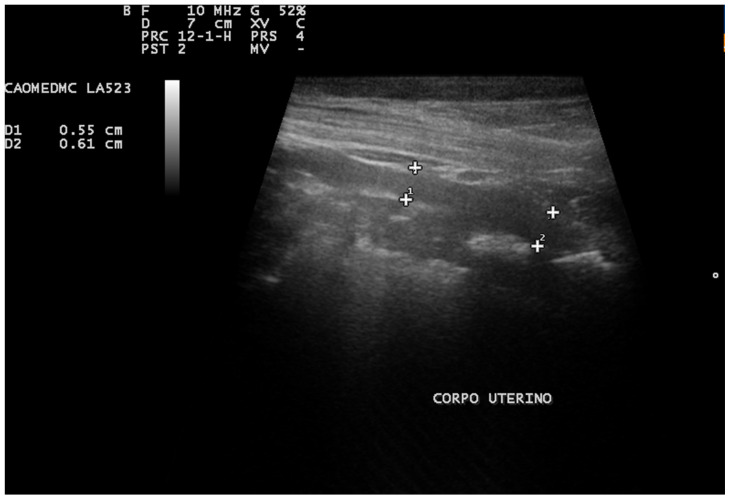
Abdominal ultrasound image of the uterus of a Pinscher. An enlarged left uterine horn measuring approximately 4.26 cm in diameter in the transverse plane is noted (cursors), with hypoechogenic content related to pyometra.

Ultrasonography has proven beneficial in identifying intrauterine fluid, even when the uterine diameter falls within the normal range (Figure 6). Additionally, it offers the advantage of revealing further pathological alterations in the tissue and ovaries, such as ovarian cysts or cystic endometrial hyperplasia [8,68].

While not commonly requested, additional complementary examinations may prove beneficial. These include histopathological analyses of the uterus following ovariohysterectomy and a microbiological culture of uterine content. These tests can confirm a diagnosis of pyometra, identify the bacteria associated with the infection, and facilitate antimicrobial susceptibility testing of the isolate [23].

## 6. Treatment

Pyometra is a medical emergency requiring prompt attention, and ovariohysterectomy (OHE) remains the preferred treatment option. Typically, a patient’s overall clinical condition reverts to normal within two weeks once the infection source is eliminated [7,9]. However, the procedure’s primary drawback is permanent sterility, which is particularly significant if the owner has a breeding interest in the animal [10]. Complications associated with OHE include hemorrhage, accidental ureteral ligation, estrogen-responsive urinary incontinence, ovarian remnant syndrome, and stump pyometra [69,70]. Stump pyometra may develop post-OHE if a section of the uterine horns or body remains and the animal exhibits elevated progesterone levels and/or an ovarian remnant [69,70,71]. The clinical manifestation, diagnosis, and treatment of stump pyometra are similar to those for pyometra, except for the history of a prior OHE [23].

While antibiotic therapy is frequently incorporated into the standard treatment protocol for pyometra, some researchers propose that perioperative antimicrobials should be reserved for animals exhibiting moderate to severe depression, thereby minimizing unnecessary antimicrobial usage [72,73]. In such instances, the initial selection of an antimicrobial should be effective against *E. coli*, the most prevalent bacteria implicated, and, ideally, adjusted based on culture and antibiogram results to a personalized narrow-spectrum alternative for each patient, thereby mitigating the risk of selecting multidrug-resistant bacteria [46,74]. However, it is worth noting that the majority of veterinarians seldom, if ever, request these culture tests [75].

Fluoroquinolones, such as enrofloxacin and amoxicillin/clavulanate, are the primary and secondary recommendations for pyometra treatment according to the Antibiotic Use Guidelines for Companion Animal Practice (Table 3) [76]. Conversely, the Finnish and Swedish guidelines propose sulfadoxine-trimethoprim and ampicillin as the preferred choices, respectively [72,77]. Research from various countries indicates that most antimicrobials, including those recommended by these guidelines, are largely effective against isolates from canine pyometra. Other effective compounds include cephalothin, streptomycin, and gentamicin [9,15,78,79,80]. A recent retrospective review corroborated these findings by demonstrating that ampicillin or amoxicillin are effective antimicrobials for cases requiring antibiotic treatment, particularly in dogs exhibiting moderate to severe general demeanor depression [73].

Pharmacological treatment has been exclusively utilized in certain scenarios, such as with young breeders or when anesthesia and surgery are currently not feasible [7,81,82]. The goal of the pharmacological management of pyometra is to actively expel purulent contents from the uterus and inhibit bacterial growth, thereby promoting uterine healing. Consequently, these protocols typically involve the simultaneous administration of steroids, antiprogestatives, and antimicrobials (Table 4). Aglepristone, a progesterone receptor blocker, and cloprostenol, a synthetic prostaglandin F_2α_ (PGF_2α_) analog, are commonly used in this regard [10,83,84]. 

In addition, antimicrobial therapy (preferably based on sensitivity tests) and supportive treatment are essential. It is important to note that these protocols are not recommended for dogs exhibiting certain clinical signs, such as fever, hypothermia, liver and/or kidney failure, or suspected peritonitis. Bitches subjected to nonsurgical treatment need to be closely monitored considering the risk of drug side effects and rapid general health deterioration, the latter of which is mostly linked to sepsis and endotoxemia. Also, owners should be aware that recurrence is possible. Interestingly, it was once believed that, following pharmacological treatment, endometrial lesions in canine pyometra would impair a dog’s ability to conceive or sustain pregnancy, but recent studies show that the pregnancy rate and litter size do not decrease [1,10,82,88,89].

## 7. Predictive Markers

Research has attempted to link prognosis with the identification of certain biomarkers [90]. Factors such as leukopenia, inappetence, azotemia, reduced packed cell volume, and dehydration have been correlated with extended hospitalization following OHE [23,64,90]. Additionally, leukopenia has been connected with the incidence of peritonitis [11,23]. 

C-reactive protein (CRP) is arguably the most extensively researched biomarker in dogs with pyometra. Current knowledge suggests that CRP levels decrease gradually following OHE, with sustained or increased concentrations potentially indicating complications [23,90,91]. Similarly, serum amyloid A, the hormone procalcitonin, and cell-free DNA exhibit the same pattern [23,90,91,92]. CRP levels are also elevated in dogs with pyometra and sepsis compared to those with mucometra [93]. Consequently, some researchers have proposed that CRP could serve as a marker for severe cases or be used to distinguish pyometra from mucometra [17,94,95]. The level of CRP has been directly linked to the length of the postoperative period [96], suggesting its potential as a valuable prognostic tool. Other studies have proposed that serum amyloid A and cell-free DNA could be used for sepsis screening, while interleukin-6 and high-mobility group Box 1 might be useful for the therapeutic monitoring of sepsis [67,90,96]. Regrettably, these biomarkers are not yet routinely used in most veterinary hospitals. Conversely, some parameters commonly included in routine testing, such as serum creatinine and urinary gamma-glutamyl transpeptidase, have not proven clinically useful in determining the severity of pyometra or renal injury in affected dogs [97].

## 8. Prevention

Elective OHE (“spaying”) serves as the primary method for pyometra prevention. However, sterilization can lead to adverse side effects, including surgical and anesthetic complications, a heightened occurrence of certain musculoskeletal and endocrinological disorders, obesity, and urinary incontinence in female dogs [98]. It is crucial to meticulously evaluate the advantages and disadvantages of such a procedure in each case, considering the breed of the animal [99,100,101].

The potential for pathogenic *E. coli* to ascend from the intestinal tract to the uterus has been documented [21,23]. This finding suggests that future research could explore how various diets influence intestinal colonization by bacteria that cause pyometra, potentially leading to preventative measures for this condition in dogs. A recent case study reported the transmission of pyometra between two Chow Chow dogs. Although the mechanisms are not fully understood, it is suggested that isolating healthy cohabiting animals from dogs with purulent vaginal discharge (indicative of open-cervix pyometra) may prevent disease transmission [22].

## 9. Future Perspectives

Canine pyometra, a potentially lethal and commonly occurring reproductive disease in female dogs, is known to be influenced by preexisting uterine lesions and hormonal and bacterial factors. However, its pathogenesis remains largely unknown. Our understanding of the etiological factors involved in *E. coli*-induced pyometra as well as the role of other pathogens is continually evolving. Future research may elucidate the influence of diet and intestinal microbiota on the risk of pyometra, potentially aiding in the development of more effective prevention protocols for this enduringly prevalent disease. Although challenging, it is necessary to determine whether certain breeds are at a higher risk of developing pyometra. Understanding the mechanisms underlying this susceptibility can help in developing novel strategies for preventing and reducing the incidence of pyometra. 

Recent studies have also described the involvement of less common pathogens in pyometra, raising the hypothesis that other infection routes, including hematogenous routes, may be more common than previously anticipated. In this context, the hypothesis that microorganisms from oral microbiota cause pyometra should be further explored.

## 10. Conclusions

Despite the significance of canine pyometra, our understanding of its epidemiology and etiopathogenesis remains limited. Pyometra is a multifactorial disease that commonly occurs during diestrus. Some established risk factors include age and the use of drugs for reproductive control. It is also likely that some breeds are predisposed to infection; however, this hypothesis has not yet been proven. Bitches with cystic endometrial hyperplasia or pseudo-placentational endometrial hyperplasia also seem to be at higher risk of developing infections. Ovariohysterectomy (OHE) remains the preferred treatment for canine pyometra; however, pharmacological treatment, commonly with anglepristone, is also possible in some specific cases.

## Figures and Tables

**Table 1:** Reported frequency of dog breeds affected by pyometra.

Breeds	Frequency (%)
Labrador Retriever	8–38
Poodle	10–33
Mixed-breed	27–30
Yorkshire Terrier	6–13
Pinscher	8–11
Golden Retriever	1–8
Rottweiler	1–8
Chow Chow	1–2
Others ^1^	<1

^1^ Other breeds include the American Pit Bull Terrier, Border Collie, German Shepherd, Lhasa Apso, Maltese, Pekingese, and Shih Tzu. Data from references [16,23,29,30,31].

**Table 2:** Most common bacterial species isolated from the uterus of female dogs with pyometra.

Organism	Frequency (%)
*Escherichia coli*	28–90
*Staphylococcus* sp.	2–42
*Klebsiella pneumoniae*	2–33
*Streptococcus* sp.	4–25
*Proteus mirabilis*	1–17
*Pseudomonas aeruginosa*	1–16
*Enterobacter* sp.	1–11
*Enterococcus* sp.	<1–3
No growth	10–26

Data from references [16,23,29,30,31].

**Table 3:** Antimicrobials recommended for the treatment of bitches with pyometra, according to published guidelines and references [72,76,77].

Drugs	Dosage	Reference
Sulfadoxine-trimethoprim	15 mg/kg/q 12 h	[72]
Ampicillin	10–20 mg/kg/q 6–8 h	[77]
Enrofloxacin	2.5–5.0 mg/kg/q 12 h	[76]
Amoxicillin/clavulanate	10–20 mg/kg/q 12 h	

**Table 4:** Examples of protocols used for pharmaceutical treatment of open cervical pyometra affecting bitches.

Drugs	Dosage	Frequency	Reference
Aglepristone	10 mg/kg q 24 h	Three doses. Days 1, 2, and 7 or 2, 7 and 14 or 1, 2 and 7	[84,85,86]
		Four doses. Days 1, 3, 6 and 9	
Aglepristone Cloprostenol	10 mg/kg q 24 h 1 μg/kg SC q 24 h	Days 1, 3, 8 and 15 Days 3 and 8	[87]

## Data Availability

No new data were created or analyzed in this study. Data sharing is not applicable to this article.
